# Better Technology, More Spending, Worse Outcomes

**DOI:** 10.5935/abc.20180055

**Published:** 2018-04

**Authors:** Whady Hueb

**Affiliations:** Instituto do Coração (InCor) do Hospital das Clínicas da Faculdade de Medicina da Universidade de São Paulo, São Paulo, SP - Brazil

**Keywords:** Myocardial Revascularization / economics, Myocardial Revascularization / mortality, Angioplasty, Balloon, Coronary, Robotic Surgical Procedures / trends, Drug-Eluting Stents / economics, Operating Rooms / trends

Since its beginning, myocardial revascularization has suffered substantial technological changes. In fact, early techniques with no physiological basis were used to increase blood supply to the ischemic myocardium. These included pericardial talc insufflation, coronary sinus ligation, Beck surgical procedure, and the Vineberg procedure. Nevertheless, due to their frustrating results that did not meet the expectations, these techniques were abandoned.

The emergence of a new, more rational technique - the coronary artery bypass surgery using venous grafts (later substituted with arterial grafts) - enabled the provision of greater blood flow to the ischemic myocardium.

Due to surgical morbidity and high costs related to material and human resources, new percutaneous techniques for coronary artery obstruction were created, including percutaneous coronary angioplasty, initially performed with balloons and then by stent therapy. In this period, intra-arterial devices and techniques such as atherotomes, Rotablator™ and laser ablation have been developed, with unsatisfactory results though. In addition, drug-eluting stents (or other stents) have been the technique of choice by interventional cardiologists. However, technological advances of these devices were accompanied by higher costs.^1^ Besides, recent studies have shown that percutaneous revascularization does not decrease cardiovascular events as compared with conventional procedures.^2,3^

In addition, with technological progresses including the use of robots and hybrid operating rooms, the number of surgery options for myocardial revascularization have increased. However, despite their refinement and safety, these techniques did not decrease the occurrence of events and cardiovascular mortality.^4^ In fact, a recent meta-analysis of nine comparative studies of revascularization surgeries performed in conventional or hybrid rooms, robot-assisted or not, indicated a worse performance of the surgeries conducted in hybrid rooms regarding event and death rates.^5^ Also, in this meta-analysis, there were disproportionate rates of reoperations (3.5%) and hemodynamic instability (9.5%) in surgeries performed in hybrid rooms, requiring the change of the surgical techniques to open procedures and extracorporeal circulation.^6^ In addition, this study showed that conventional surgery had a better revascularization performance as compared with the technique performed in hybrid rooms. However, it is worth mentioning that the efficacy of complete and incomplete myocardial revascularization is still a matter of debate. Studies comparing the efficacy of complete, incomplete or no revascularization showed similar results between the procedures.^7^

Finally, 40 years has passed since the publication of the CASS Trial,^8^ which pointed out that regardless of the number and extension of arteries involved, clinical and surgical therapy have comparable results in patients with preserved ventricular function and stable angina, with an annual mortality rate of approximately 2%. Therefore, in the CASS Trial,^8^ considering that clinical therapy was based only in the use of beta-blockers and prolonged-action nitrates, one may consider that the surgery was compared with a control group (placebo).
